# Agreement between self-reported and registered age at asthma diagnosis in Finland

**DOI:** 10.1186/s12890-024-02949-3

**Published:** 2024-03-15

**Authors:** Elias Nurmi, Iida Vähätalo, Pinja Ilmarinen, Heidi Andersén, Leena E. Tuomisto, Anssi Sovijärvi, Helena Backman, Lauri Lehtimäki, Linnea Hedman, Arnulf Langhammer, Bright I. Nwaru, Päivi Piirilä, Hannu Kankaanranta

**Affiliations:** 1 Tampere University Respiratory Research group, Faculty of Medicine and Health Technology, Tampere University, Tampere, Finland; 2 Department of Respiratory Medicine, Seinäjoki Central Hospital, Seinäjoki, Finland; 3 Oncology Unit, Vaasa Keskussairaala, Vaasa, Finland; 4 Faculty of Medicine, University of Helsinki, Helsinki, Finland; 5 Department of Clinical Physiology and Nuclear Medicine, Unit of Clinical Physiology, HUS Medical Imaging Center, Helsinki University Central Hospital and University of Helsinki, Helsinki, Finland; 6 Department of Public Health and Clinical Medicine, Section of Sustainable Health, The OLIN Unit, Umeå, Sweden; 7 Allergy Centre, Tampere University Hospital, Tampere, Finland; 8 Department of Public Health and Nursing, HUNT Research Centre Faculty of Medicine and Health Sciences, NTNU, Norwegian University of Science and Technology, Levanger, Norway; 9 Levanger Hospital, Nord-Trøndelag Hospital Trust, Levanger, Norway; 10 Krefting Research Centre, Institute of Medicine, Dept of Internal Medicine and Clinical Nutrition, Sahlgrenska Academy, University of Gothenburg, Gothenburg, Sweden; 11 Wallenberg Centre for Molecular and Translational Medicine, University of Gothenburg, Gothenburg, Sweden

**Keywords:** Asthma, Age at diagnosis, Questionnaire, Health register, Agreement, Reliability

## Abstract

**Introduction:**

In epidemiological studies, the age at asthma onset is often defined by patients’ self-reported age at diagnosis. The reliability of this report might be questioned. Our objective was to evaluate the agreement between self-reported and registered age at asthma diagnosis and assess features contributing to the agreement.

**Methods:**

As part of the FinEsS respiratory survey in 2016, randomly selected population samples of 13,435 from Helsinki and 8000 from Western Finland were studied. Self-reported age at asthma diagnosis was compared to age at asthma diagnosis registered in the Finnish register on special reimbursement for asthma medication. The reimbursement right is based on lung function criteria according to GINA and Finnish guidelines. If the difference was less than 5 years, self-reported diagnosis was considered reliable. Features associated with the difference between self-reported and registered age at asthma diagnosis were evaluated.

**Results:**

Altogether 197 subjects from Helsinki and 144 from Western Finland were included. Of these, 61.9% and 77.8%, respectively, reported age at diagnosis reliably. Median difference between self-reported and registered age at diagnoses was − 2.0 years (IQR − 9.0 to 0) in Helsinki and − 1.0 (IQR − 4.3 to 0) in Western Finland indicating earlier self-reported age at diagnosis. More reliable self-report was associated with non-allergic subjects and subjects who reported having asthma diagnosis more recently.

**Conclusions:**

Agreement between self-reported and registered age at asthma diagnosis was good especially with adult-onset asthma patients. Poor agreement in early-onset asthma could be related to delay in registration due to reimbursement criteria.

**Supplementary Information:**

The online version contains supplementary material available at 10.1186/s12890-024-02949-3.

## Highlights

Self-reported age at asthma diagnosis was compared with health register data.

Agreement between self-report and register was good in adult-onset asthma.

If the diagnosis was reported far in the past, agreement with register was poorer.

## Introduction

Asthma is a heterogeneous chronic respiratory disease, which affects > 300 million people of all ages worldwide [1]. Diagnosis of asthma is based on variable respiratory symptoms and variable expiratory airflow limitation usually measured with pulmonary function tests. However, there is no unambiguous diagnostic standard for asthma [1, 2]. 

Asthma is often considered as mainly ailment of young children which becomes less common with increasing age. However, recent epidemiologic studies from Finland and the USA suggest that adult-onset asthma is common, especially among women aged 30–35 years and older [3–5]. Increasing knowledge on asthma phenotypes has shown that age at asthma onset is an important consideration in phenotypic categorization [6–8]. Age at asthma diagnosis helps in phenotyping asthma since allergic asthma more often begins in childhood or adolescence. Age at asthma onset considerably affects asthma prognosis and remission. Early-onset asthma often remits in adolescence, unlike adult-onset asthma, which seldom remits. Thus, the knowledge of age at onset is crucial in evaluating the prognosis of asthma, as well as research on causal factors and factors influencing the disease development [8–12]. Furthermore, the age at diagnosis is required for estimation of incidence, cumulative prevalence and point prevalence.

Self-reported year of asthma diagnosis has been previously suggested to be reliable when evaluated against a prior interview with a nurse [13] and against answers from identical questionnaires [14, 15]. However, those studies relied entirely on self-reported information. A different way to assess the reliability of self-reported age at asthma diagnosis would be to compare it with health register data. This is possible in Finland as there are population-wide health registers and asthma diagnosis is based on objective pulmonary function tests [3, 16] except in children under 3–5 years of age. Further, the date of asthma diagnosis can be derived from the registers.

The aim of this study was to evaluate the agreement between self-reported and registered age at asthma diagnosis. To the best of our knowledge, there are no previous studies on this topic.

## Methods

### Data acquisition, FinEsS questionnaire and the Nordic EpiLung study

The present study was conducted as a part of FinEsS (Finland, Estonia, Sweden) study and the Nordic EpiLung study consortium, an epidemiologic respiratory research collaboration project between Finland, Sweden, and Norway.

**Fig. 1:**
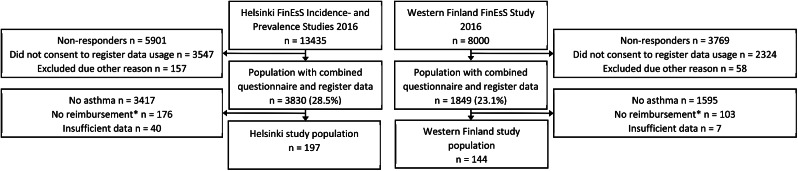
Flowchart of the study. *Special asthma medication reimbursement granted by the Finnish Social Insurance Institution. Reimbursement represents registered asthma diagnosis

**Fig. 2:**
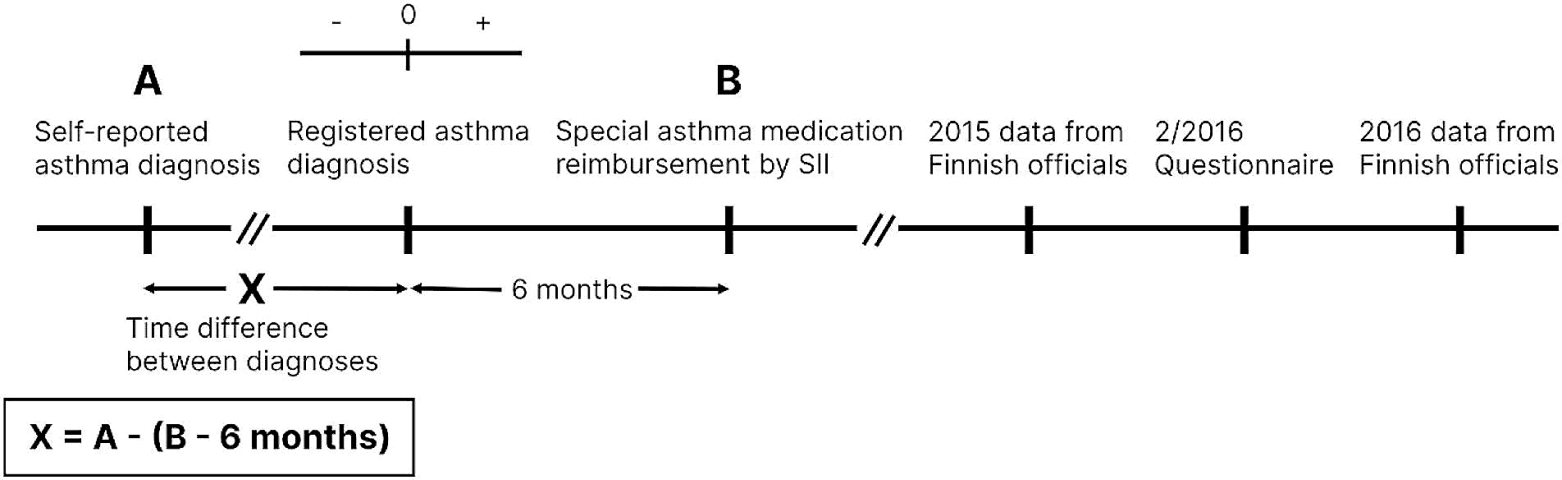
Definition of time difference between diagnoses and notable timepoints visualized in an event diagram. SII = Finnish Social Insurance Institution

As a part of the cross-sectional FinEsS survey, a postal respiratory questionnaire was sent to randomly selected cohorts in Helsinki and Western Finland areas in February 2016. The FinEsS questionnaire is developed from the OLIN questionnaire as previously described [5, 17, 18]. In Helsinki, a total of 13,435 persons were invited to the FinEsS study. The Helsinki cohort consists of the Helsinki FinEsS Incidence Study (follow-up of a cohort surveyed in 1996) and -Prevalence Study (new cohort in 2016). Similarly in Western Finland, 8000 persons were invited to the Western Finland FinEsS Study from the Hospital Districts of Vaasa and Seinäjoki. The total study population size was 341 persons with registered and self-reported physician-diagnosed asthma. (Fig. 1) More detailed flowcharts are presented in supplementary material (S-Figs. 1 and 2).

The selection of the original study populations was conducted randomly by Statistic Finland in 10-year age cohorts, matching gender distribution to local populations. Study participants were 19–89 years of age.

For the study participants who gave permission to use their register data, the FinEsS questionnaire data was combined with comprehensive data provided by Finnish government agencies: Statistics Finland (STAT), The Social Insurance Institution (SII) and The Finnish Tax Administration. These government agencies provided official register data on demographics, medication purchases and medication reimbursements for years 2015 and 2016. By using unique personal identification number, the questionnaire data was combined with the data provided by these agencies.

### Applying medication reimbursement data

The Finnish medication reimbursement system was introduced in 1964 by The Health Insurance Act. It is a government-funded initiative, enforced by the SII. Its purpose is to financially support patients who require constant medication to treat physician diagnosed diseases. The SII has listed the reimbursed diseases, each with specific diagnostic criteria for reimbursement entitlement. In the case of asthma, the reimbursement covers 65% of the cost of their medication to treat asthma [3, 19, 20]. After receiving an asthma diagnosis, fulfilling at least one of the various diagnostic lung function criteria and after continuously using anti-inflammatory medication for a minimum of six months, the patients may apply for the special asthma medication reimbursement. Those with intermittent medication or poor adherence may not meet the reimbursement criteria. For children under 16 years, the reimbursement is granted for a limited period, and it must be reapplied. For over 16-year-olds, the reimbursement is granted permanently. In the cases with reissued reimbursements, e.g., first in childhood and later in adulthood, the date of the initial entitlement is given in the register. More detailed description of the SII requirements for special asthma medication reimbursement is presented in the supplementary material (S-Table 1).

In this study, we used the dates of special asthma medication reimbursements to estimate the registered age at asthma diagnosis. Given the requirement of the six-month continuous asthma therapy, we defined the age at diagnosis as follows: If the reimbursement was issued in the second half of the year (July to December), age in the reimbursement year was used. For entitlements issued in the first half of the year, the age from the previous year was used.

### Comparing questionnaire- and register data

Self-reported physician-diagnosed asthma and age at diagnosis were defined by the questions “Have you been diagnosed by a doctor as having asthma?” and “What age were you when asthma was diagnosed?”, respectively.

Time difference between diagnoses, i.e., the difference between self-reported age at diagnosis and registered age at diagnosis was calculated by subtracting the registered age at diagnosis from the self-reported age at diagnosis (Fig. 2).

Good agreement between self-reported and registered age at diagnosis was defined as difference of − 5 to 5 years. This definition was made in compliance with incidence studies where age is often categorized in 5- or 10-year age groups [3, 4, 21]. Data from the registers together with the FinEsS questionnaire were used to assess features associated with the time difference between diagnoses.

### Definitions of other key study variables

*Allergic rhinitis* was defined by a positive answer to: “Have you been diagnosed by a doctor as having allergic rhinitis by pollen (caused by, e.g. birch, grass, mugwort)?” or “Have you been diagnosed by a doctor as having other allergic rhinitis (caused by, e.g. cat or dog, but not caused by pollen)?” *Allergic conjunctivitis* was defined by a positive answer to: “Have you been diagnosed by a doctor as having symptoms of allergy in your eyes?” *Allergic dermatitis* was defined by a positive answer to: “Do you have an itchy rash diagnosed by a doctor as infantile atopic dermatitis, Besnier’s prurigo or atopic eczema?” Definitions of the rest of the study variables are presented in the supplementary material.

### Statistical analyses

Mann-Whitney U test and t-test were used for continuous variables as appropriate. Fisher’s exact test (expected cell counts of < 5) and Pearson’s chi-squared test (χ^2^) were used for categorical variables. Data are presented as median and IQR, mean and SD, numbers (n) and percentage (%).

Reliability of self-reported age at diagnosis, i.e., the ability of self-report to give same values for age at diagnosis as register despite the measurement error was measured with two-way mixed effects, absolute agreement, single measurement intraclass correlation coefficient (ICC) [22]. ICC was interpreted as instructed by Koo & Li [23]. Agreement between self-reported and registered age at diagnosis, i.e., the closeness of self-reported and registered age at diagnosis was shown with nonparametric Bland-Altman plot [22]. Nonparametric variation was chosen because the differences did not follow normal distribution. Bias was represented with median, and limits of agreement were represented with 5th and 95th percentiles [24]. If possible, 95% confidence intervals were plotted. Acceptance limit of − 5 to 5 years was used for limits of agreement. A p-value < 0.05 was considered statistically significant.

Statistical analyses were performed using R (version 4.2.2 for Helsinki data and version 3.6.2 for Western Finland data, R Foundation for Statistical Computing, Vienna, Austria). The ICC was calculated using IBM SPSS Statistics (version 28 for Helsinki data and version 27 for Western Finland data, Armonk, NY, USA).

The Western Finland data was accessible only within FIONA remote access system (Statistics Finland) and thus the Western Finland data and Helsinki data could not be combined and were analyzed independently. According to the data safety rules of Statistics Finland, datapoints with 3 or less participants are not given and *n* ≤ 3 is shown instead for the Western Finland data. Missing data contributed less than 5% across all variables.

## Results

### Helsinki and Western Finland cohorts

Helsinki and Western Finland populations were mostly similar with exceptions in Body Mass Index (BMI), sex and prevalence of allergic rhinitis (Table 1).

**Table 1:** Basic characteristics of the whole population (asthma and non-asthma) and characteristics of the study population (with asthma) in Helsinki and Western Finland

Whole population				
	Helsinki (*n* = 3830)		Western Finland (*n* = 1849)	
	Median	IQR	Median	IQR
Age (years) - Male - Female	55 56 55	44–65 45–65 44–65	56 57 54	42–63 44–63 40–62
	**N**	**%**	**N**	**%**
Female	2164	56.8	973	52.6
**Study population**				
	**Helsinki (n = 197)**		**Western Finland (n = 144)**	
	**Median**	**IQR**	**Median**	**IQR**
Age (years) - Male - Female	59.0 59.0 59.0	50.0-68.0 45.5-68.0 50.0-66.2	59.0 62.0 58.0	45.8–65.0 48.0–66.0 41.0–65.0
BMI (kg/m^2^) - Male - Female	26.0 25.7 26.1	23.0-29.7 23.6-28.8 22.8-30.1	27.9 28.7 27.4	24.4–31.5 25.6–33.0 23.2–30.3
	**N**	**%**	**N**	**%**
Female	132	67.7	83	57.6
Smoking - Current - Ex - Never	33 76 88	16.8 38.6 44.7	22 56 65	15.4 39.2 45.5
	**Median**	**IQR**	**Median**	**IQR**
**Age at asthma diagnosis (years)**				
Time elapsed from self-reported asthma diagnosis	19.0	9.0–28.0	18.0	8.8–26.0
Time elapsed from registered asthma diagnosis	12.0	6.0–21.0	13.0	6.0–21.0
Self-reported age at asthma diagnosis	40.0	29.0–51.0	40.0	24.8–50.0
	**N**	**%**	**N**	**%**
Self-reported age at asthma diagnosis - <12 - 12–39 - ≥40	17 76 104	8.6 38.6 52.8	14 55 75	9.7 38.2 52.1
**Medication**				
History of one or more R03 purchases	183	92.9	129	89.6
Use of asthma medication	186	94.4	131	91.0
	**Median**	**IQR**	**Median**	**IQR**
**Allergies, symptoms, and allergic heredity**:				
Physician-diagnosed allergic rhinitis	102	51.8	64	44.4
Family history of asthma	97	49.2	73	50.7
**Asthma symptoms**:				
Attacks of breathlessness now or during the last 12 months	152	77.2	114	79.2
Wheeze last 12 months	113	57.4	84	58.3
Tightness in the chest last 12 months	93	47.2	62	43.1
Hospitalization or visits to emergency department due to asthma exacerbation during last 12 months	17	8.6	12	8.3

IQR, interquartile range; BMI, Body Mass Index; R03, ATC code for drugs for obstructive airway diseases; COPD, Chronic obstructive pulmonary disease

### Overall agreement between self-reported and registered age at asthma diagnosis

**Fig. 3:**
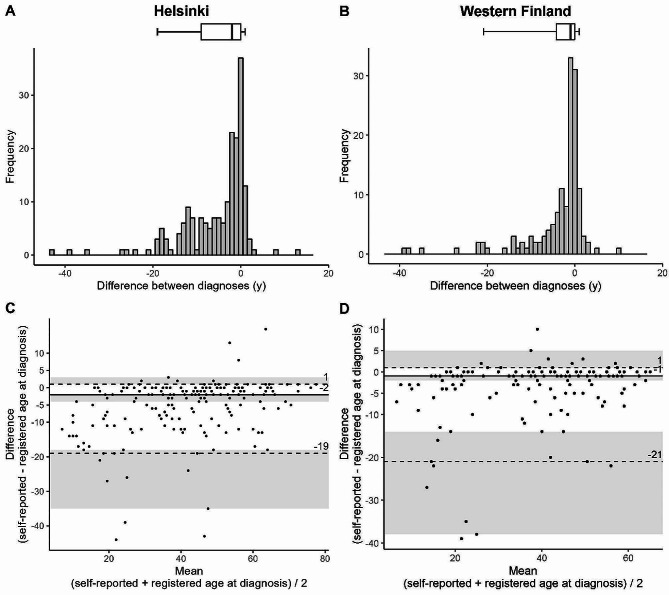
Time difference between diagnoses and Bland-Altman plot in Helsinki (**A**, **C**) and in Western Finland (**B**, **D**). In boxplots (**A**, **B**), median and IQR is shown. Whiskers represent 5th and 95th percentiles. The distribution of time difference is shown in the histograms (**A**, **B**). Negative values indicate earlier self-reported age at diagnosis. In Bland-Altman plots (**C**, **D**), solid line (━) represents median, and the dashed lines (- - -) represent limits of agreement (90% of the measured values). Shaded area represents 95% confidence intervals

The median time difference between self-reported and registered diagnoses was − 2.0 years (IQR − 9.0 to 0) in Helsinki and − 1.0 years (IQR − 4.3 to 0) in Western Finland indicating earlier self-reported age at diagnosis. In Helsinki, 61.9% reported age at asthma diagnosis within 5 years from the registered asthma diagnosis, viewed as reliable, compared to 77.8% in Western Finland. The intraclass correlation (ICC) between self-reported and registered age at asthma diagnosis was 0.826 (95% CI, 0.593–0.909) in Helsinki study population and 0.866 (95% CI, 0.746–0.922) in Western Finland indicating moderate to excellent reliability. According to nonparametric Bland-Altman plot between self-reported and registered age at diagnosis, limits of agreement (LoA) was [− 19.0, 1.0] and bias was − 2.0 years in Helsinki. The corresponding LoA was [− 20.9, 1.0] and bias was − 1.0 years in Western Finland. Both lower limits of agreement exceed the − 5 year lower acceptance limit viewed as reliable but both upper limits of agreement are well within the 5 year acceptance limit. (Fig. 3; Table 2)

**Table 2:** Bland Altman analysis and intraclass correlation coefficients between self-reported and registered age at asthma diagnosis

	Helsinki			Western Finland		
	Total	Lack of allergy	Self-report within 10 years	Total	Lack of allergy	Self-report within 10 years
Bland-Altman agreement: Bias and [LoA] in years	−2.0 [− 19.0, 1.0]	−1.0 [− 14.9, 1.0]	−1.0 [− 2.5, 1.0]	−1.0 [− 20.9, 1.0]	−1.0 [− 8.1, 1.0]	0 [− 4.0, 1.0]
Intraclass correlation: ICC with [95% CI]	0.826 [0.593, 0.909]	0.897 [0.756, 0.933]	0.989 [0.980, 0.994]	0.866 [0.746, 0.922]	0.947 [0.883, 0.972]	0.991 [0.982, 0.996]

LoA, limits of agreement; ICC, intraclass correlation coefficient; CI, confidence interval

There was no significant difference in the agreement between male and female responders (S-Tables 2 and 3).

### Comparison of participants with good and poor agreement between self-reported and registered age at asthma diagnosis

To understand the features associated with the difference between self-reported and registered age at asthma diagnosis, the asthma patients were first divided in two groups according to the difference between self-reported and registered ages at diagnosis: good agreement (≤ 5 years) and poor agreement (> 5 years). Subjects with poor agreement reported asthma diagnosis 11.5 to 17 years further in the past and at 13 to 15 years younger age compared to those with good agreement (Table 3).

**Table 3:** Comparison of age at asthma diagnosis of subjects with good* and poor** agreement

	Helsinki					Western Finland				
	Good agreement*: *n* = 122		Poor agreement**: *n* = 75			Good agreement*: *n* = 112		Poor agreement**: *n* = 32		
Age at asthma diagnosis (years)	Median	IQR	Median	IQR	P	Median	IQR	Median	IQR	P
Time elapsed from self-reported asthma diagnosis	11.0	6.3–21.8	28.0	21.0-35.5	< 0.001	15.0	7.0–23.0	26.5	19.0-33.2	< 0.001
Time elapsed from registered asthma diagnosis	10.5	5.3–19.8	15.0	9.0-24.5	0.023	13.5	5.8–22.0	12.0	6.0-18.2	0.429
Self-reported age at asthma diagnosis	45.0	35.0–55.0	30.0	14.0–40.0	< 0.001	43.0	29.5–52.0	30.0	7.8–40.0	< 0.001
	**N**	**%**	**N**	**%**		**N**	**%**	**N**	**%**	
Self-reported age at asthma diagnosis										
- < 12	1	0.8	16	21.3	< 0.001	4	3.6	10	31.2	< 0.001
- 12–39	42	34.4	34	45.3		43	38.4	12	37.5	
- ≥ 40	79	64.8	25	33.3		65	58.0	10	31.2	

*Good agreement: self-reported age at asthma diagnosis within − 5 to 5 years from the registered age at asthma diagnosis

**Poor agreement: self-reported age at asthma diagnosis further than − 5 or 5 years from the registered age at asthma diagnosis

IQR, interquartile range

With subjects with < 10 years elapsed from self-reported asthma diagnosis, the median time difference between self-reported and registered age at diagnosis in Helsinki and Western Finland was − 1.0 (IQR − 1.0 to 0) and 0 (IQR − 1.0 to 0) years, respectively. Conversely, the median difference was − 4 to − 2 years greater in participants with ≥ 10 years elapsed from self-reported diagnosis as compared to participants with < 10 years elapsed from self-reported diagnosis. Similar association was seen when time difference was categorized into good and poor agreement. (Table 4)

**Table 4:** Difference between diagnoses in subjects with shorter* and longer** time elapsed from self-reported asthma diagnosis

	Helsinki					Western Finland				
	< 10 years elapsed from self-reported asthma diagnosis		≥ 10 years elapsed from self-reported asthma diagnosis		P	< 10 years elapsed from self-reported asthma diagnosis		≥ 10 years elapsed from self-reported asthma diagnosis		P
Variable	Median	IQR	Median	IQR		Median	IQR	Median	IQR	
Difference between self-reported and registered diagnoses (years)	−1.0	−1.0 to 0	−5.0	−12.0 to − 1.0	< 0.001	0	−1.0 to 0	−2.0	−6.5 to − 0.5	< 0.001
	**N**	**%**	**N**	**%**		**N**	**%**	**N**	**%**	
Categorized difference between self-reported and registered diagnoses										
- Good agreement (− 5 to 5 years)	50	98.0	72	49.3	< 0.001	40	97.6	72	69.9	< 0.001
- Poor agreement ( < − 5 or > 5 years)	1	2.0	74	50.7		≤ 3	≤ 7.3	31	30.1	

*<10 years elapsed from self-reported asthma diagnosis

**≥10 years elapsed from self-reported asthma diagnosis

IQR, interquartile range

Across all subjects, the prevalence of self-reported asthma medication use was over 84% and over 89% had a minimum of one recorded asthma medication purchase in the register. Allergic symptoms were more prevalent in subjects with poor agreement between self-report and register (*p* < 0.05). Also, the prevalence of wheeze was higher with those who had poor agreement in Helsinki (*p* < 0.05). (Table 5; S-Tables 2, 3 and 4).

**Table 5:** Comparison of respiratory symptoms and -heredity of subjects with good* and poor** agreement

	Helsinki					Western Finland				
	Good agreement*: *n* = 122		Poor agreement**: *n* = 75			Good agreement*: *n* = 112		Poor agreement**: *n* = 32		
	**N**	**%**	**N**	**%**	**P**	**N**	**%**	**N**	**%**	**P**
**Physician-diagnosed allergy**:										
Allergic rhinitis	49	40.2	53	70.7	< 0.001	44	39.3	20	62.5	0.033
Allergic rhinitis due to pollen	38	31.1	44	58.7	< 0.001	37	33.0	17	53.1	0.062
Allergic rhinitis due to other factors	34	27.9	41	54.7	< 0.001	26	23.2	16	50.0	0.007
Allergic conjunctivitis	32	26.2	32	42.7	0.025	25	22.3	11	34.4	0.247
Allergic dermatitis	25	20.5	20	26.7	0.408	21	18.8	15	46.9	0.003
**Heredity**:										
Family history of asthma	56	45.9	41	54.7	0.295	56	50.0	17	53.1	0.911
Family history of allergic rhinitis or conjunctivitis	49	40.2	42	56.0	0.044	47	42.0	12	37.5	0.803
**Asthma symptoms**:										
Attacks of breathlessness now or during the last 12 months	90	73.8	62	82.7	0.204	92	82.1	22	68.8	0.162
Longstanding cough during the last 12 months	47	38.5	25	33.3	0.560	43	38.4	12	37.5	1.000
Sputum production	62	50.8	41	54.7	0.705	74	66.1	15	46.9	0.078
Periods of sputum production for at least 2 consecutive years	31	25.4	21	28.0	0.815	33	29.5	7	21.9	0.534
Recurrent wheeze	30	24.6	33	44.0	0.007	36	32.1	9	28.1	0.829
Wheeze last 12 months	61	50.0	52	69.3	0.012	69	61.6	15	46.9	0.198
Tightness in the chest last 12 months	54	44.3	39	52.0	0.363	50	44.6	12	37.5	0.605
Dyspnea mMRC ≥ 2	45	36.9	39	52.0	0.053	47	42.0	14	43.8	1.000
Hospitalization or visits to emergency department due to asthma exacerbation during last 12 months	9	7.4	8	10.7	0.591	10	8.9	≤ 3	≤ 9.4	1.000

*Good agreement: self-reported age at asthma diagnosis within − 5 to 5 years from the registered age at asthma diagnosis

**Poor agreement: self-reported age at asthma diagnosis further than − 5 or 5 years from the registered age at asthma diagnosis

COPD, Chronic obstructive pulmonary disease; mMRC, Modified Medical research council dyspnea scale

In Helsinki, 27 (13.7%) and in Western Finland, 21 (14.6%) reported having physician-diagnosed chronic bronchitis, chronic obstructive pulmonary disease (COPD) or emphysema in addition to asthma. The prevalence of these comorbidities did not affect to the agreement between self-reported and registered age at asthma diagnosis (data not shown).

### Features associated with agreement between self-reported and registered diagnosis

**Fig. 4:**
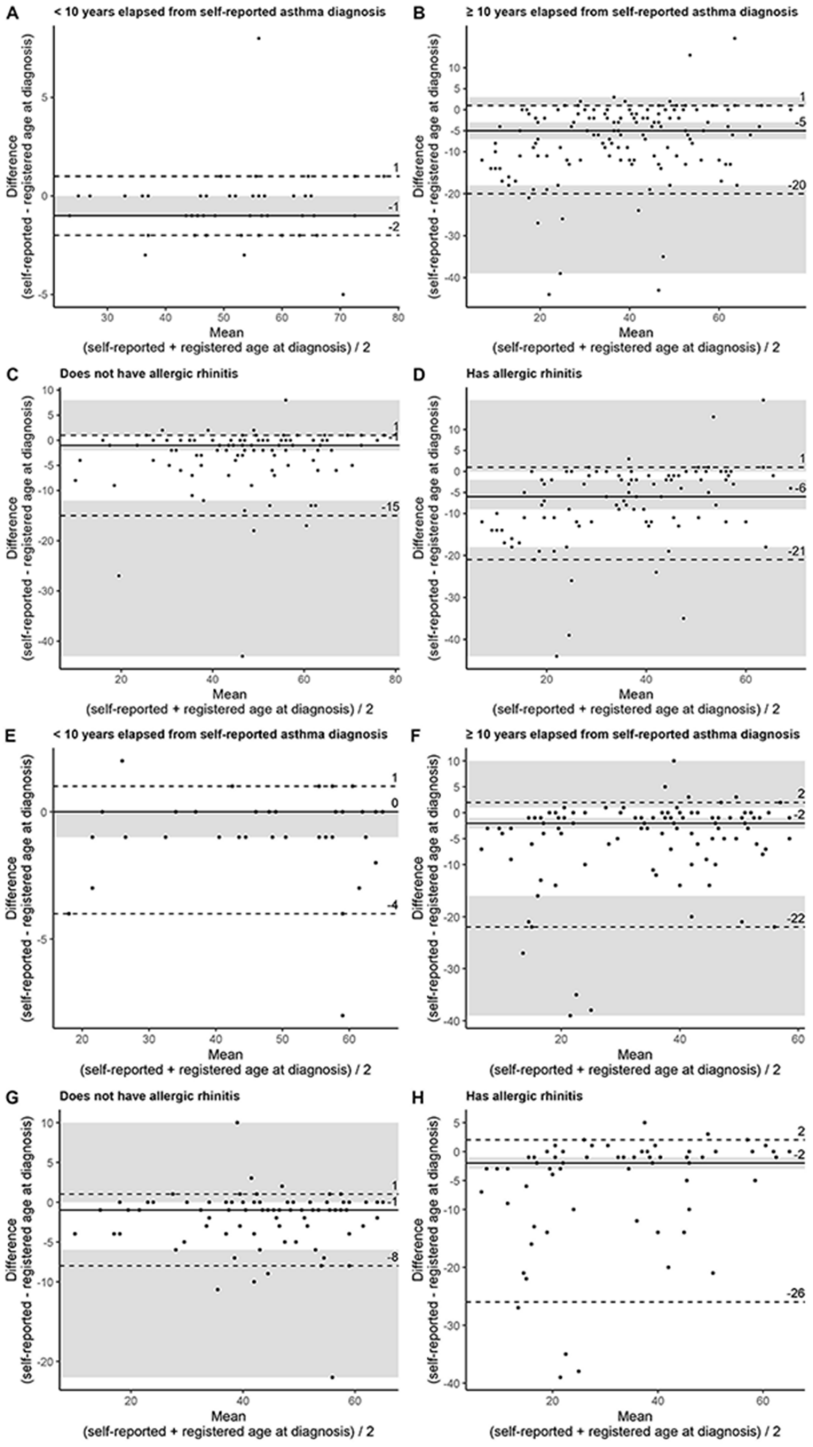
Bland-Altman plots of agreement between self-reported and registered age at diagnosis from Helsinki (**A**–**D**) and Western Finland (**E**–**H**) study populations. (**A**,**E**) population with less than 10 years elapsed from self-reported asthma diagnosis, (**B**,**F**) population with 10 or more years elapsed from self-reported asthma diagnosis, (**C**, **G**) population without allergic rhinitis, (**D**, **H**) population with allergic rhinitis. Solid line (━) represents median, and the dashed lines (- - -) represent limits of agreement (90% of the measured values). Shaded area represents 95% confidence intervals. In (**A**, **E**, **G**, **H**) all 95% CI:s could not be calculated

Subjects who reported having asthma diagnosis more recently and subjects without allergic symptoms tended to report age at diagnosis more reliably (Tables 4 and 5). This was further affirmed with agreement analysis (Bland-Altman plots) and reliability analysis (intraclass correlation coefficient, ICC). Intraclass correlation showed good to excellent reliability, with lower limit of the 95% CI being 0.756 to 0.982. Equally, Bland-Altman analysis indicated mainly good agreement, with upper limit of agreement being 1.0 in all analyses. The lower limit of agreement was − 4.0 to − 2.5 years in subjects with recently reported diagnosis, recently meaning within the last 10 years, and − 14.9 to − 8.1 years in subjects without allergy. The acceptance limits were defined as − 5 and 5 years. (Table 2; Fig. 4) More detailed description of the Bland-Altman analysis and intraclass correlation is shown in the supplementary material.

The correlations between years elapsed from self-reported physician diagnosis and time difference between diagnoses were (*r* = − 0.54, *P* < 0.001; *r* = − 0.46, *P* < 0.001) in Helsinki and Western Finland, showing moderate correlation. The correlations between years elapsed from registered diagnosis and time difference between diagnoses and the correlations between age and time difference between diagnoses did not show significance. (S-Figs. 3 and 4).

## Discussion

### Main findings

According to our study, the majority (61.9 − 77.8%) of asthma patients had good agreement between self-reported and registered age at asthma diagnosis. The portion of reliable responses was higher in asthma patients who reported having asthma diagnosis more recently or did not report allergic symptoms. On average, Bland-Altman analysis and intraclass correlation coefficient between self-reported and registered age at asthma diagnosis showed good agreement.

### Features associated with reliability of self-reported age at asthma diagnosis

In the current study, poor agreement was associated with questionnaire results indicating a long time ago diagnosed asthma and allergic tendency. These features can be a result of the same cause, as early-onset asthma is generally associated with an allergic component [6, 7]. In adults, the allergic asthma may have had onset a long time ago in childhood, teenage or early adulthood [25]. Also, allergic asthma may develop gradually from atopic dermatitis and food allergy in a process referred as atopic march, causing ambiguity in perceived disease onset [26, 27]. We assume that there is no direct causal connection between the presence of allergic symptoms and the agreement between self-reported and registered ages at diagnosis. Yet, the presence of allergic symptoms implies an earlier asthma onset or slow progression of the disease in some patients and is associated with longer time elapsed from self-reported diagnosis.

Our findings suggest that some people may overestimate the elapsed time from diagnosis and report the age at diagnosis being at a younger age compared to the age at diagnosis derived from the health register. Similar deviation was found in a Swedish ten-year follow-up study [13]. This would partially explain the poorer observed agreement in adults with features of early-onset asthma. Suggestions of recall bias due to long time elapsed from diagnosis and old age have been made previously [28]. 

However, we did not find a significant relationship between bias and age. Additionally, study design is reported to influence the perceived incidence of asthma, which may reflect to the perceived age at asthma onset. A longer follow-up time may underestimate the incidence and vice versa as described in a Swedish longitudinal study [29]. 

### Validating asthma diagnosis in epidemiologic studies

Questionnaire-based asthma diagnosis has been previously validated against follow-up questionnaires, interviews, clinical diagnosis, or clinical examinations e.g., bronchodilatation, methacholine or histamine challenge tests. However, follow-up questionnaires and interviews rely completely on self-reported data, and do not provide an objective reference point. Clinical diagnosis often relies on self-report and empiric symptom-based assessment, instead of objective lung function tests. Despite limitations, validating the diagnosis by a follow-up study has been found highly reliable, supporting our findings [13–15].

### Challenges of asthma diagnosis in epidemiologic studies globally and in Finland

Globally, the applied diagnostic guidelines for asthma are mainly consensus based and not completely evidence based. The present diagnostic criteria have limitations and could be improved [30, 31]. The limitations in asthma diagnosis may have affected to the completeness of the health registers. Traditionally in several countries, asthma diagnosis is based solely on physician’s empiric assessment of symptoms and objective pulmonary function tests are not always conducted. Generally, in register studies, all subjects with asthma diagnostic code in the register are included in the study, without considering the diagnostic basis of asthma.

The GINA has established distinct guidelines for diagnosing asthma which are not all fully utilized worldwide [32–34]. In Finland, independent, evidence-based, GINA compliant clinical practice guidelines known as Current Care Guidelines are followed. These guidelines for asthma diagnosis are uniform with the criteria set for special asthma medication reimbursement entitlement by the Social Insurance Institution of Finland [2, 3, 16]. Consequently, in Finland the registered asthma diagnoses are largely correct, but we cannot exclude underdiagnosis.

In defining asthma for this study, we established a criterion of having special asthma medication reimbursement. However, the reimbursement is not a direct indicator of an asthma diagnosis. Subjects entitled to the reimbursement can be reliably considered to have asthma, but conversely, the absence of the reimbursement does not necessarily indicate the absence of asthma, as asthma medication purchases are an essential part of reimbursement decision.

### Causes of delay in asthma diagnosis and reimbursement

The registered asthma diagnosis date may be inaccurate for some subjects. This inaccuracy can occur because the required six-month continuous use of asthma medication, a prerequisite for special asthma medication reimbursement, might be completed over six months from the physician’s diagnosis date. This delay often results from irregular use of asthma medication or poor adherence to the prescribed treatment. Subjects with mild or intermittent asthma may receive treatment for asthma but are not entitled to special asthma medication reimbursement due to intermittent therapy. This is relevant especially among atopic subjects. Later if asthma progresses, these subjects may be qualified for the reimbursement, and this potentially results in large time difference between self-reported and registered diagnoses. Delay in the physician-diagnosis may also result from the possibility that sufficient diagnostic findings are not found on the initial examination due to suboptimal sensitivity of spirometry even though asthma symptoms are present [31]. Some delay may result from the duration from onset of asthma symptoms to earliest constellation of symptoms. In children this duration was found short with median duration being widely under one year [35].

If the special asthma medication reimbursement has been granted more than once e.g., first in childhood and again after resurfacing in adulthood, the self-report may refer to the latest reimbursement while only the first granted reimbursement is denoted in the register. This may cause significant bias towards larger (positive) time difference between self-reported and registered diagnoses for small portion of the subjects, possibly explaining some outliers.

The potential bias in the time difference between diagnoses resulting from the delay of the reimbursement is small in most cases and does not affect our study significantly as reliable self-report was defined within − 5 to 5 years from the registered diagnosis.

### Limitations of health registers and retrospective study design

According to a Finnish study, health registers may contain faulty data which is also a possible source of errors [36]. The amount of faulty data on diagnosis in the Finnish health registers is estimated to be approximately 5% [37]. The amount is minor in comparison to 12–30% of faulty data on asthma diagnosis in countries where the asthma diagnosis does not require objective pulmonary function tests [38–40]. As the Finnish medication reimbursement system begun in 1964, some subjects may have had asthma diagnosis before the existence of reimbursement registers, resulting in bias. According to self-reports < 3% of subjects reported physician-diagnosis of asthma occurring before 1964. These subjects were excluded from the analysis.

Suboptimal response rate of 54.7% combined with exclusion of participants who denied permission to use their register data is a limitation of the study. The total 26.6% of study invitees who gave permission to use their register data may be more conscientious compared to an average citizen, which can cause bias. Furthermore, potential incorrect questionnaire responses and interpretation errors may account to some bias.

### Significance and applications

In epidemiological studies on asthma phenotypes, reliable data on age at asthma onset is essential. According to our study, age at physician-diagnosis of asthma obtained from questionnaire-based studies is predominantly a viable and reliable variable. This is especially important for epidemiological studies related to asthma pathogenesis and phenotypes. However, if the self-reported date or age at diagnosis is far in the past, the responses have poorer agreement with register data, as expected. In contrast, self-reported age at diagnosis with adult-onset asthma patients seem reliable.

Including age at asthma diagnosis to surveys may result to more accurate data on asthma diagnosis, compared to only asking if asthma is diagnosed. This might improve the quality of future questionnaire studies on asthma prevalence and incidence.

## Conclusions

According to our study, the reliability of self-reported age at physician diagnosis of asthma is good. Agreement between self-reported and registered age at asthma diagnosis is high especially in patients with characteristics of adult-onset asthma. Our study confirms the use of questionnaire-based age at diagnosis in asthma studies regarding adult-onset asthma patients.

### Electronic supplementary material

Below is the link to the electronic supplementary material.

Supplementary Material 1

Supplementary Material 2

Supplementary Material 3

Supplementary Material 4

Supplementary Material 5

## Data Availability

The datasets generated and/or analyzed during the current study are not publicly available due to confidential nature of the data and data safety rules. Further inquiries about the data can be directed to Dr. Hannu Kankaanranta.

## Abbreviations

IQR: Interquartile range
FinEsS: Finland-Estonia-Sweden study
OLIN: Obstructive Lung Disease in Northern Sweden questionnaire
STAT: Statistics Finland
SII: The Social Insurance Institution of Finland
ICC: Intraclass Correlation Coefficient
R03: ATC-code for Drugs for Obstructive Airway Diseases
mMRC: Modified Medical Research Council dyspnea scale
LoA: Limits of Agreement
GINA: Global initiative for asthma
