# Supplementary figures and images for: Agreement between self-reported and registered age at asthma diagnosis in Finland
Source: BMC Pulm Med. 2024 Mar 15;24:133. doi: 10.1186/s12890-024-02949-3 (PMC10943976; doi:10.1186/s12890-024-02949-3)

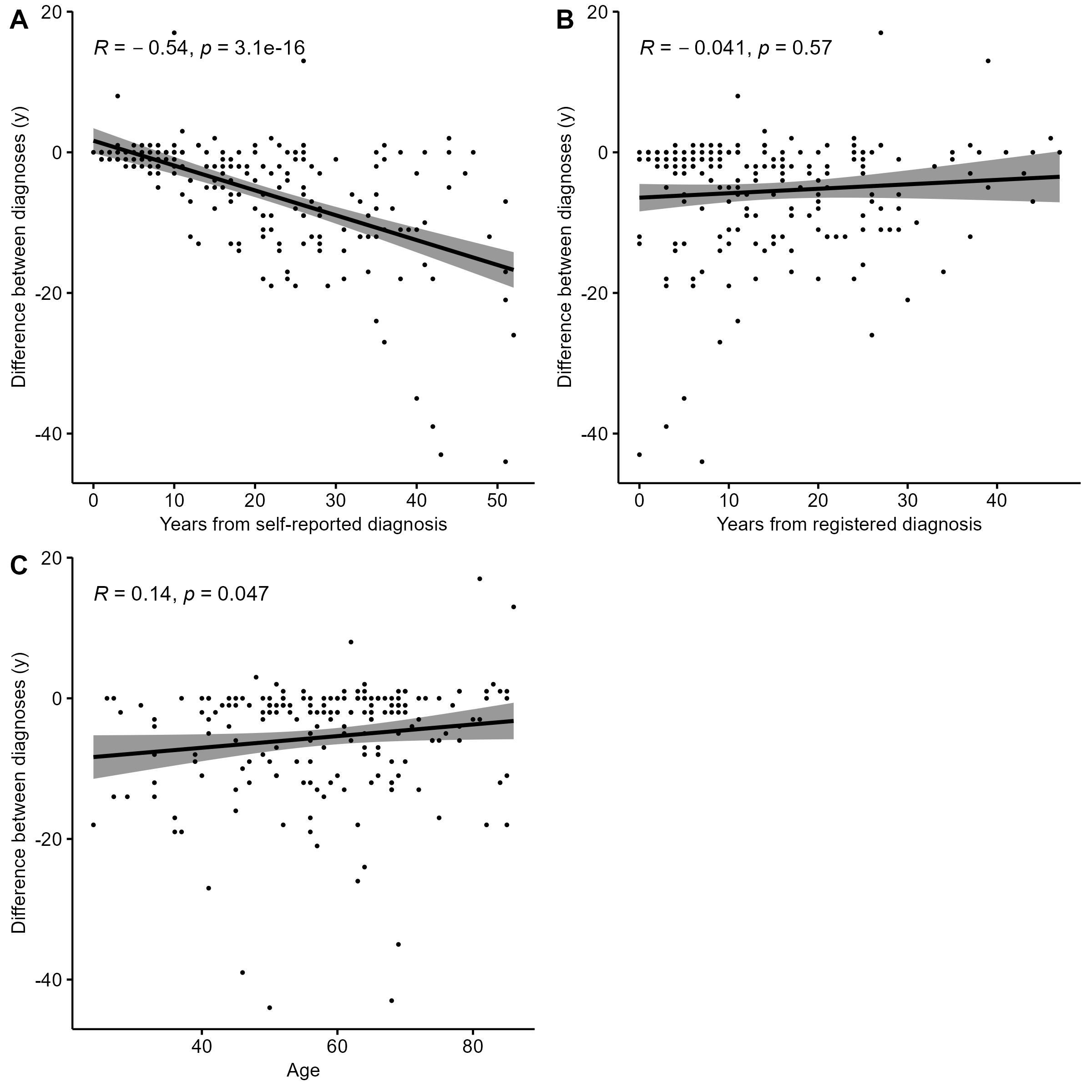

Supplement: Supplementary file 1 — Supplementary Material 1 [file 12890_2024_2949_MOESM1_ESM.jpg]

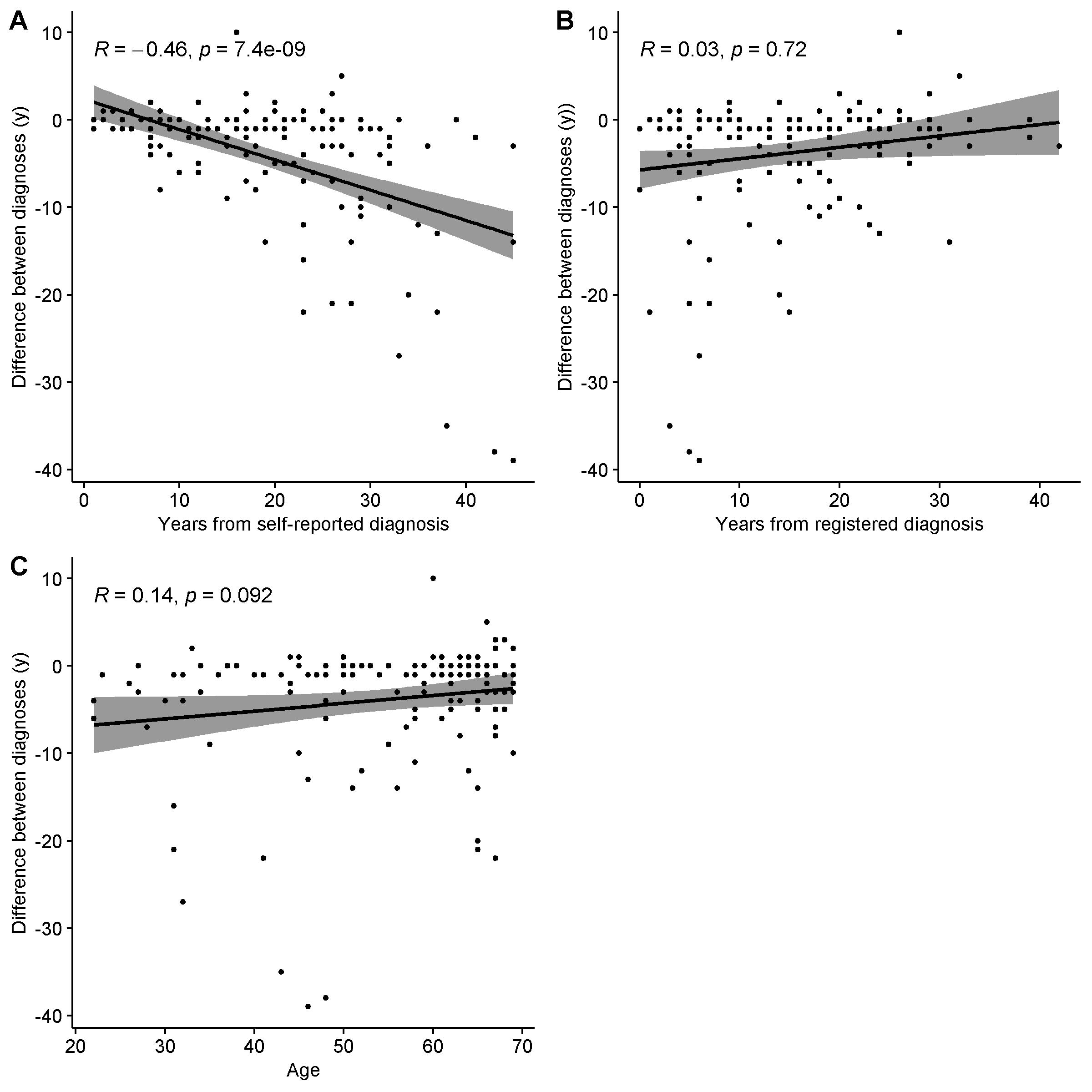

Supplement: Supplementary file 2 — Supplementary Material 2 [file 12890_2024_2949_MOESM2_ESM.jpg]

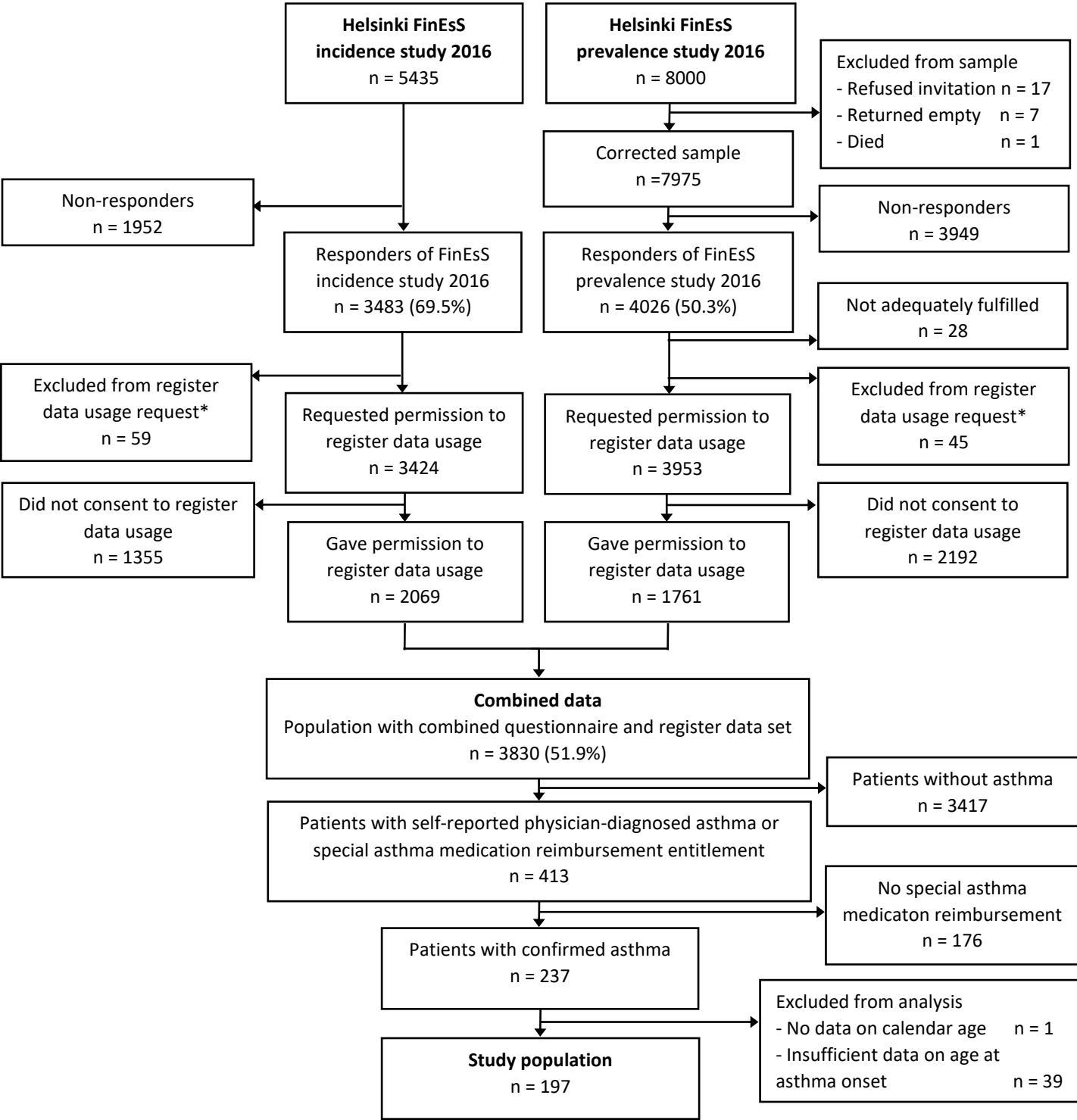

Supplement: Supplementary file 3 — Supplementary Material 3 [file 12890_2024_2949_MOESM3_ESM.pdf]

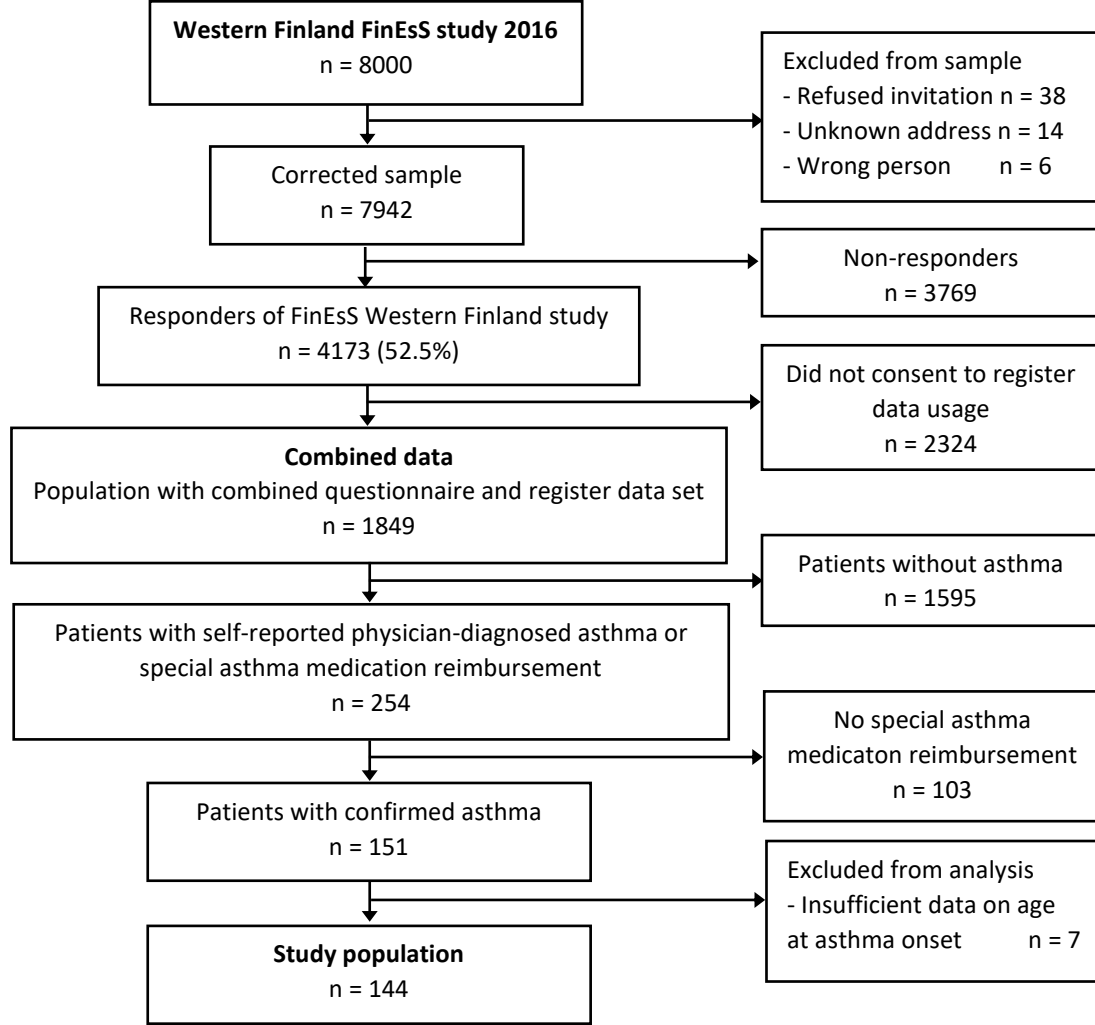

Supplement: Supplementary file 4 — Supplementary Material 4 [file 12890_2024_2949_MOESM4_ESM.pdf]
