# Supplementary material for: Agreement between self-reported and registered age at asthma diagnosis in Finland
Source: BMC Pulm Med. 2024 Mar 15;24:133. doi: 10.1186/s12890-024-02949-3 (PMC10943976; doi:10.1186/s12890-024-02949-3)
Supplement: Supplementary file 5 — Supplementary Material 5 [file 12890_2024_2949_MOESM5_ESM.docx]

**SUPPLEMENTARY MATERIAL**

**Agreement between self-reported and registered age at asthma diagnosis in Finland**

Elias Nurmi^a,b^, Iida Vähätalo^a,b^, Pinja Ilmarinen^a,b^, Heidi Andersén^a,c^, Leena E. Tuomisto^a,b^, Anssi Sovijärvi^d,e^, Helena Backman^f^, Lauri Lehtimäki^a,g^, Linnea Hedman^f^, Arnulf Langhammer^h,i^, Bright I. Nwaru^j,k^, Päivi Piirilä^d,e,*^, Hannu Kankaanranta^a,b,j,*^

^a^Tampere University Respiratory Research group, Faculty of Medicine and Health Technology, Tampere University, Tampere, Finland
^b^Department of Respiratory Medicine, Seinäjoki Central Hospital, Seinäjoki, Finland
^c^Oncology Unit, Vaasa Keskussairaala, Vaasa, Finland
^d^Faculty of Medicine, University of Helsinki, Helsinki, Finland
^e^Unit of Clinical Physiology, Department of Clinical Physiology and Nuclear Medicine, HUS Medical Imaging Center, Helsinki University Central Hospital and University of Helsinki, Helsinki, Finland
^f^Department of Public Health and Clinical Medicine, Section of Sustainable Health, The OLIN Unit, Umeå, Sweden
^g^Allergy Centre, Tampere University Hospital, Tampere, Finland
^h^HUNT Research Centre, Department of Public Health and Nursing, Faculty of Medicine and Health Sciences, NTNU, Norwegian University of Science and Technology, Levanger, Norway
^i^Levanger Hospital, Nord-Trøndelag Hospital Trust, Levanger, Norway
^j^Krefting Research Centre, Institute of Medicine, Dept of Internal Medicine and Clinical Nutrition, Sahlgrenska Academy, University of Gothenburg, Gothenburg, Sweden
^k^Wallenberg Centre for Molecular and Translational Medicine, University of Gothenburg, Gothenburg, Sweden
*equal contribution

**Declaration of interests:** Dr. Ilmarinen is employed by GSK. Dr. Tuomisto reports personal fees for lectures from GSK. Dr. Backman reports personal fees for lectures form AstraZeneca, Boehringer Ingelheim and GSK. Dr. Lehtimäki reports personal fees for lectures and consulting from ALK, AstraZeneca, Boehringer Ingelheim, Chiesi Pharma, GSK, MSD, Novartis, Orion Pharma and Sanofi Genzyme. Dr Langhammer reports personal fees for lectures and consulting from Boehringer Ingelheim, GSK, AstraZeneca and Diagnostica Ltb. Dr. Nwaru reports personal fees for lectures and consulting from DBV Technologies and AstraZeneca. Dr. Kankaanranta reports personal fees for lectures and consulting from AstraZeneca, BoehringerIngelheim, Chiesi Pharma, GSK, MSD, Novartis, Orion Pharma and Sanofi Genzyme. The other authors declare no conflict of interest related to this study.

**Supplementary methods**

**Definitions of all other parameters**

*History of one or more R03 purchases* was defined by a record of one or more purchases of drugs for obstructive airway diseases (ATC code R03) within years 2015 or 2016. Records were provided by the Finnish Social Insurance Institution (SII) and cover all pharmacies in Finland. Drugs under R03 are not available “over-the-counter” in Finland and thus the data represents all purchases of R03.
*Use of asthma medication* was defined by a positive response to: “Do you currently use asthma medicines permanently or as needed?” in the FinEsS questionnaire.
*Allergic rhinitis due to pollen* was defined by a positive answer to: “Have you been diagnosed by a doctor as having allergic rhinitis by pollen (caused by, e.g. birch, grass, mugwort)?” in the FinEsS questionnaire.
*Allergic rhinitis due other factor* was defined by a positive answer to: “Have you been diagnosed by a doctor as having other allergic rhinitis (caused by, e.g. cat or dog, but not caused by pollen)?” in the FinEsS questionnaire.
*Family history of asthma* was defined by a positive answer to: “Have any of your parents, brothers or sisters now or previously had asthma?” in the FinEsS questionnaire.
*Family history of allergic rhinitis or -conjunctivitis* was defined by a positive answer to: “Have any of your parents, brothers or sisters had allergic eye-/nose catarrh (hay-fever)?” in the FinEsS questionnaire.
*Family history of chronic bronchitis, COPD or emphysema* was defined by a positive answer to: “Have any of your parents, brothers or sisters had chronic bronchitis or emphysema?” in the FinEsS questionnaire.
*Attacks of breathlessness now or during the last 10 years* was defined by a positive answer to: “Have you now or have you had asthma symptoms during the last 10 years (intermittent breathlessness) or attacks of breathlessness? The symptoms may exist simultaneously with or without cough or wheezing.” in the FinEsS questionnaire.
*Attacks of breathlessness now or during the last 12 months* was defined by a positive answer to: “Have you had intermittent breathlessness or attacks of breathlessness, with or without simultaneously appearing cough or wheezing during the last 12 months” in the FinEsS questionnaire.
*Longstanding cough during the last 12 months* was defined by a positive answer to: “Have you had longstanding cough during the last year?” in the FinEsS questionnaire.
*Sputum production* was defined by a positive answer to: “Do you usually have phlegm when coughing, or do you have phlegm which is difficult to bring up?” in the FinEsS questionnaire.
*Sputum production most of the days during periods lasting at least 3 months* was defined by a positive answer to: “Do you bring up phlegm on most days during periods of at least successive three months? ” in the FinEsS questionnaire.
*Periods of sputum production for at least 2 consecutive years* was defined by a positive answer to: “Have you had such periods during at least two successive years?” referring to the previous question in the FinEsS questionnaire.
*Recurrent wheeze* was defined by a positive answer to: “Have you had wheezing, whistling, or a noisy sound in your chest when breathing?” in the FinEsS questionnaire.
*Wheeze last 12 months* was defined by a positive answer to: “Have you had wheezing or whistling in your chest at any time in the last 12 months?” in the FinEsS questionnaire.
*Wheeze with dyspnea* was defined by a positive answer to: “Have you been at all breathless when the wheezing noise was present?” referring to the previous question in the FinEsS questionnaire.
*Wheeze without cold* was defined by a positive answer to: “Have you had this wheezing or whistling when you did not have a cold?” referring to “Wheeze last 12 months” in the FinEsS questionnaire.
*Tightness in the chest last 12 months* was defined by a positive answer to: “Have you awakened with a feeling of tightness in your chest at any time in the last 12 months?” in the FinEsS questionnaire.
*Dyspnea mMRC ≥2* was defined by a positive answer to: “Do you have to walk slower than other people of your age on level ground because of breathlessness?” in the FinEsS questionnaire.
*Visits to emergency department due to asthma exacerbation during last 12 months* was defined by a positive answer to: “Have you had to visit emergency department because of an asthma attack in the last year?” in the FinEsS questionnaire.
*Hospitalization due asthma exacerbation during last 12 months* was defined by a positive answer to: “Have you been hospitalized due asthma exacerbation in the last year?” in the FinEsS questionnaire.
*Hospitalization or visits to emergency department due to asthma exacerbation during last 12 months* was defined by a positive answer to either of the two previous questions in the FinEsS questionnaire.

**Supplementary results**

**Description of the Bland-Altman plots and intraclass correlation coefficients in the sub-analyses**

The ICC between self-reported and registered age at diagnosis for study participants with less than 10 years elapsed from self-reported diagnosis was 0.989 (95% CI 0.980 – 0.994) in Helsinki and 0.991 (95% CI 0.982 – 0.996) in Western Finland indicating excellent reliability. For study participants with 10 or more years elapsed from self-reported diagnosis, the ICC was 0.768 (95% CI 0.373 – 0.891) in Helsinki indicating poor to good reliability and 0.789 (95% CI 0.562 – 0.885) in Western Finland indicating moderate to good reliability. According to nonparametric Bland-Altman plots between self-reported and registered age at asthma diagnosis for study participants with less than 10 years elapsed from self-reported diagnosis, the limits of agreement (LoA) was [−2.5, 1.0] and bias was −1.0 years in Helsinki. The corresponding LoA was [−4.0, 1.0] and bias was 0 years in Western Finland. Both LoAs fit in the set −5 to 5 year acceptance limits. For study participants with 10 or more years elapsed from self-reported diagnosis, the LoA was [−20.5, 1.0] and bias was −5.0 years in Helsinki. The corresponding LoA was [−21.9, 1.9] and bias was −2.0 years in Western Finland. The lower limit of agreement exceeds the −5 year lower acceptance limit viewed as reliable, but the upper limit of agreement is well within the 5 year acceptance limit.

The ICC for study participants with reported allergic rhinitis was 0.752 (95% CI 0.349 – 0.884) in Helsinki indicating poor to good reliability and 0.776 (95% CI 0.517 – 0.885) in Western Finland indicating moderate to good reliability. For non-allergic study participants, the ICC was 0.897 (95% CI 0.756 – 0.933) in Helsinki and 0.947 (95% CI 0.883 – 0.972) in Western Finland indicating good to excellent reliability. According to nonparametric Bland-Altman plots for study participants with reported allergic rhinitis, LoA was [−20.9, 1.0] and bias was −5.5 years in Helsinki. The corresponding LoA was [−26.3, 1.9] and bias was −2.0 year in Western Finland. For non-allergic study participants, LoA was [−14.9, 1.0] and bias was −1.0 years in Helsinki. The corresponding LoA was [−8.1, 1.0] and bias was −1.0 in Western Finland. Both lower limits of agreement exceed the −5 year lower acceptance limit viewed as reliable but both upper limits of agreement are well within the 5 year acceptance limit.

These results are visualized in Table 2 and Figure 4 in the main article.

**Supplementary tables**

**S-Table 1.** Criteria for special asthma medication reimbursement in Finland according to Social Insurance Institution of Finland.

| Asthma is considered as a chronic disease and applicable to special medication reimbursement entitlement in children and adults when the regular anti-inflammatory medication has continued at least 6 months and still continues. | |
| --- | --- |
| Diagnostic basis for chronic asthma in adults and in children ≥7 year of age. | - Diurnal variation in the peak expiratory flow (PEF) during 2-week follow-up of at least 20% as compared with the mean of morning and evening values at least 3 times. - During PEF follow-up, PEF-value increases in response to bronchodilator repeatedly for at least 3 times ≥15 % (in adults, also ≥60 l/min) as compared with the pre-bronchodilator value. - In the bronchodilatation test made with spirometry, forced expiratory volume in one second (FEV_1_) or forced vital capacity (FVC) increases ≥12 % and ≥200 ml as compared with the pre-bronchodilator value. - In a therapeutic trial with corticosteroids FEV_1_ increases ≥15 % and ≥200 mL or the mean PEF-values during several days (preferably 5 days before medication as compared with the 5 last days of the therapeutic trial) increase ≥20%. - In bronchial provocation test (either histamine or methacholine), the bronchial hyperreactivity is moderate to severe. - In exercise-test PEF or FEV_1_ is reduced ≥15 % from pre-trial value. |
| Diagnostic basis for chronic asthma in children <7 years of age. | - Asthma in children below school-age (less than 7 years of age) should be confirmed with lung function tests. Evaluation of lung function tests is usually successful after the age of 5-6 years. The results of the lung function tests must be documented. Asthma diagnosis in children aged less than 3 years is based on symptoms and clinical findings. In small children in whom the diagnosis is based on symptoms and clinical findings, features suggesting asthma are expiratory wheezing and breathing difficulty that occur repeatedly and relieve of symptom with bronchodilatory medication. Features supporting the diagnosis of asthma are need for the bronchodilatory medication more than twice a week and breathing difficulties that require increased medication and which occur with an interval less than six weeks. - In small children with physician-diagnosed breathing difficulties 2-3 times a year, a clinical index based on asthma risk factors can be used. In those cases, the probability of asthma is increased when at least one primary criterion or 2 secondary criteria are met.   Primary criteria:   1. Parental asthma, either father or mother 2. Physician-diagnosed atopic dermatitis 3. Immunoglobulin E (IgE) -mediated food allergy   Secondary criteria:   1. Physician-diagnosed allergic rhinitis 2. Wheezing in the absence of infection 3. Proportion of blood eosinophilic granulocytes >4%.   If breathing difficulties exist at least 4 times a year, a regular anti-inflammatory medication reducing bronchial inflammation is advised. In small children, variable obstruction found using special methods (e.g., oscillometry) or elevated exhaled nitric oxide content reflecting eosinophilic airway inflammation found using special methods are additional information that can complement the evaluation. |
| Entitlement to special asthma medication reimbursement in children as well as in adults requires the use of anti-inflammatory medication that reduces airway inflammation at least for 6 months. In children aged less than 16 years of age, special asthma medication reimbursement can be granted for a period of 5 years at maximum and in children less than 3 years of age, special asthma medication reimbursement can be granted for a period of 2 years at maximum. | |

Note. Modified from “Age-specific incidence of new asthma diagnoses in Finland” by Kankaanranta H, Tuomisto LE, Ilmarinen P. *J allergy Clin Immunol Pract*. 2017;5(1):189-191.e3. doi:10.1016/j.jaip.2016.08.015

**S-Table 2.** Comparison of characteristics of subjects with good* and poor** agreement in Helsinki study population.

|  | Good agreement*: n = 122 | | Poor agreement**:  n = 75 | |  |
| --- | --- | --- | --- | --- | --- |
| **Variable** | **Mean** | **SD** | **Mean** | **SD** | **P** |
| Age (years)  - Male  - Female | 58.7  57.5  59.2 | 13.0  16.1  11.4 | 57.7  57.5  56.7 | 14.8  17.1  13.0 | 0.633  0.999  0.266 |
|  | **Median** | **IQR** | **Median** | **IQR** |  |
| BMI (kg/m^2^)  - Male  - Female | 26.1  27.1  26.0 | 22.9-29.6  23.6-28.9  22.4-29.7 | 25.9  25.4  27.2 | 23.3-29.9  23.6-28.0  23.2-30.5 | 0.789  0.419  0.338 |
|  | **N** | **%** | **N** | **%** |  |
| Female | 83 | 68.0 | 49 | 67.1 | 1.000 |
| Smoking  - Current  - Ex  - Never | 17  46  59 | 13.9  37.7  48.4 | 16  30  29 | 21.3  40.0  38.7 | 0.278 |
| **Medication** |  |  |  |  |  |
| History of one or more R03 purchases | 115 | 94.3 | 68 | 90.7 | 0.504 |
| Use of asthma medication | 116 | 95.1 | 70 | 93.3 | 0.751 |

*Good agreement: self-reported age at asthma diagnosis within −5 to 5 years from the registered age at asthma diagnosis

**Poor agreement: self-reported age at asthma diagnosis further than −5 or 5 years from the registered age at asthma diagnosis

SD, standard deviation; IQR, interquartile range; BMI, Body Mass Index; R03, ATC code for drugs for obstructive airway diseases

**S-Table 3.** Comparison of characteristics of subjects with good* and poor** agreement in Western Finland study population.

|  | Good agreement*:  n = 112 | | Poor agreement**: n = 32 | |  |
| --- | --- | --- | --- | --- | --- |
|  | **Median** | **IQR** | **Median** | **IQR** | **P** |
| Age (years)  - Male  - Female | 60.5  63.0  58.5 | 46.8-66.0  50.0-66.0  42.5-65.2 | 55.5  57.0  52.0 | 44.5-64.2  46.0-63.0  38.0-64.5 | 0.195  0.230  0.451 |
| BMI (kg/m^2^)  - Male  - Female | 28.2  28.6  27.6 | 24.6-31.8  25.7-33.1  23.7-30.7 | 27.6  29.4  26.1 | 23.5-30.3  25.3-32.1  22.9-28.6 | 0.788  0.891  0.676 |
|  | **N** | **%** | **N** | **%** |  |
| Female | 64 | 57.1 | 19 | 59.4 | 0.982 |
| Smoking  - Current  - Ex  - Never | 16  46  49 | 14.4  41.4  44.1 | 6  10  16 | 18.8  31.2  50.0 | 0.563 |
| **Medication** |  |  |  |  |  |
| History of one or more R03 purchases | 100 | 89.3 | 29 | 90.6 | 1.000 |
| Use of asthma medication | 104 | 92.9 | 27 | 84.4 | 0.164 |

*Good agreement: self-reported age at asthma diagnosis within −5 to 5 years from the registered age at asthma diagnosis

**Poor agreement: self-reported age at asthma diagnosis further than −5 or 5 years from the registered age at asthma diagnosis

SD, standard deviation; IQR, interquartile range; BMI, Body Mass Index; R03, ATC code for drugs for obstructive airway diseases

**S-Table 4.** Difference between diagnoses in subjects with and without allergy

|  | Difference between self-reported and registered diagnoses (years) | |  | Difference between self-reported and registered diagnoses (years) | |  |
| --- | --- | --- | --- | --- | --- | --- |
| **Helsinki** | | | | | | |
|  | **Median** | **IQR** |  | **Median** | **IQR** | **P** |
| Allergic rhinitis | −5.5 | −12.0 to −1.0 | No allergic rhinitis | −1.0 | −5.0 to 0 | <0.001 |
| Allergic conjunctivitis | −5.5 | −11.2 to −1.0 | No allergic conjunctivitis | −2.0 | −7.0 to 0 | 0.022 |
| Allergic rhinitis or allergic conjunctivitis | −5.5 | −12.0 to −1.0 | No allergic rhinitis or allergic conjunctivitis | −1.0 | −5.0 to 0 | <0.001 |
| Allergic rhinitis and allergic conjunctivitis | −7.5 | −12.0 to −1.2 | No allergic rhinitis and allergic conjunctivitis | −2.0 | −7.0 to 0 | 0.007 |
| **Western Finland** | | | | | | |
| Allergic rhinitis | −2.0 | −10.0 to 0 | No allergic rhinitis | −1.0 | −3.2 to 0 | 0.063 |
| Allergic conjunctivitis | −2.0 | −9.8 to 0 | No allergic conjunctivitis | −1.0 | −4.0 to 0 | 0.232 |
| Allergic rhinitis or allergic conjunctivitis | −2.0 | −9.8 to 0 | No allergic rhinitis or allergic conjunctivitis | −1.0 | −3.0 to 0 | 0.015 |
| Allergic rhinitis and allergic conjunctivitis | −1.5 | −10.8 to 0 | No allergic rhinitis and allergic conjunctivitis | −1.0 | −4.0 to 0 | 0.572 |

IQR, interquartile range

**Supplementary figures**

**Helsinki FinEsS incidence study 2016**
n = 5435

**Helsinki FinEsS prevalence study 2016**
n = 8000

Responders of FinEsS incidence study 2016
n = 3483 (69.5%)

Responders of FinEsS prevalence study 2016
n = 4026 (50.3%)

Corrected sample
n =7975

Requested permission to register data usage
n = 3424

Requested permission to register data usage
n = 3953

Gave permission to register data usage
n = 2069

Gave permission to register data usage
n = 1761

**Combined data**
Population with combined questionnaire and register data set
n = 3830 (51.9%)

Patients with self-reported physician-diagnosed asthma or special asthma medication reimbursement entitlement
n = 413

Patients with confirmed asthma
n = 237

**Study population**
n = 197

Excluded from sample
- Refused invitation n = 17
- Returned empty n = 7
- Died n = 1

Non-responders
n = 3949

Not adequately fulfilled
n = 28

Excluded from register data usage request*
n = 45

Did not consent to register data usage
n = 2192

Patients without asthma
n = 3417

No special asthma medicaton reimbursement
n = 176

Excluded from analysis
- No data on calendar age n = 1
- Insufficient data on age at asthma onset n = 39

Non-responders
n = 1952

Excluded from register data usage request*
n = 59

Did not consent to register data usage
n = 1355

**S-Figure 1.** Flowchart of the study: Helsinki. *Exclusion includes faulty signatures, empty forms, denied consent etc.

**Western Finland FinEsS study 2016**
n = 8000

Corrected sample
n = 7942

Responders of FinEsS Western Finland study
n = 4173 (52.5%)

**Combined data**
Population with combined questionnaire and register data set
n = 1849

Patients with self-reported physician-diagnosed asthma or special asthma medication reimbursement
n = 254

Patients with confirmed asthma
n = 151

**Study population**
n = 144

Excluded from sample
- Refused invitation n = 38
- Unknown address n = 14
- Wrong person n = 6

Non-responders
n = 3769

Did not consent to register data usage
n = 2324

Patients without asthma
n = 1595

No special asthma medicaton reimbursement
n = 103

Excluded from analysis
- Insufficient data on age at asthma onset n = 7

**S-Figure 2.** Flowchart of the study: Western Finland


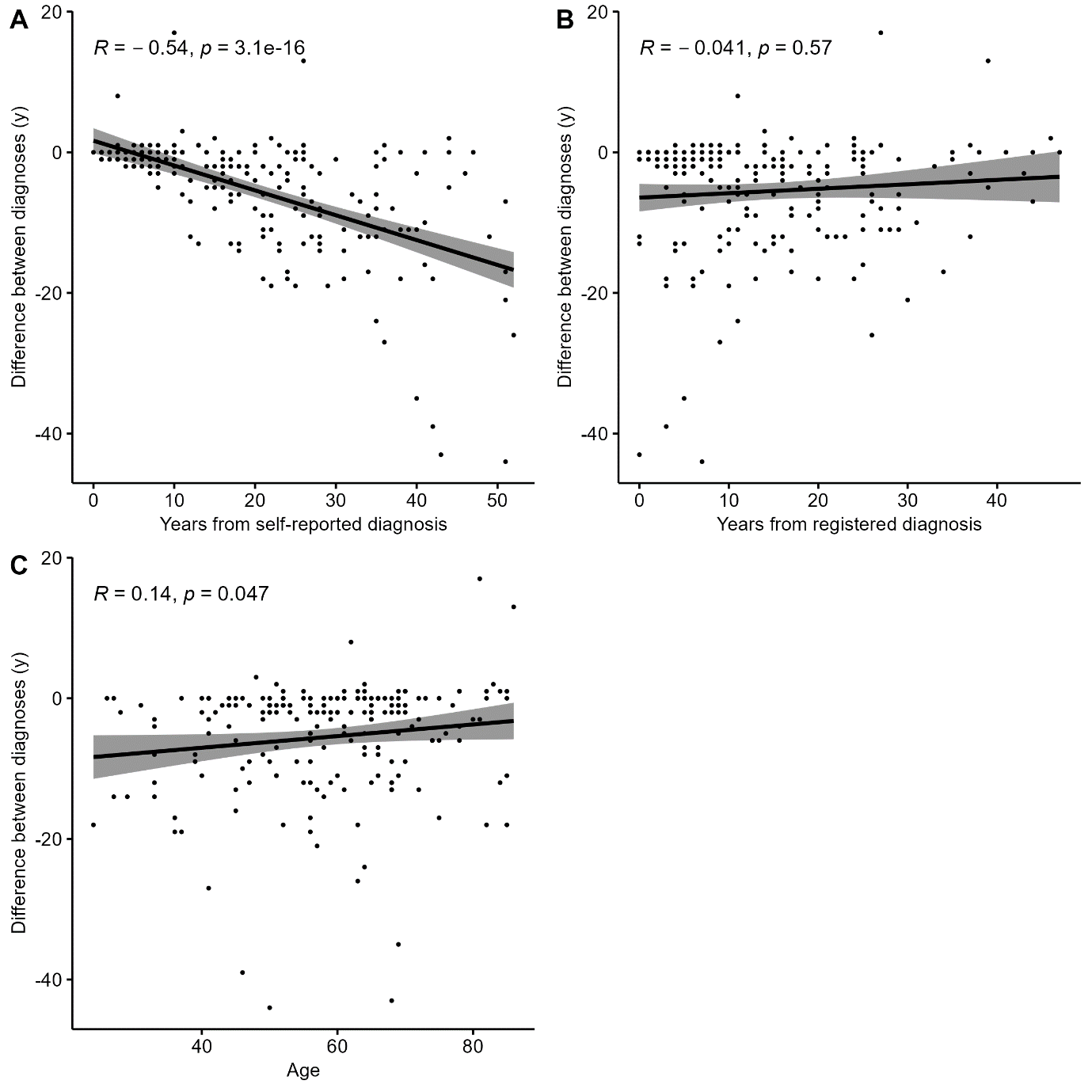
 **S-Figure 3.** Correlation between the time difference and years elapsed from self-reported physician-diagnosed asthma (A), between the time difference and years elapsed from registered asthma diagnosis (B) between the time difference and calendar age (C) in Helsinki. Spearman’s *r* and P-value of the correlation is shown.


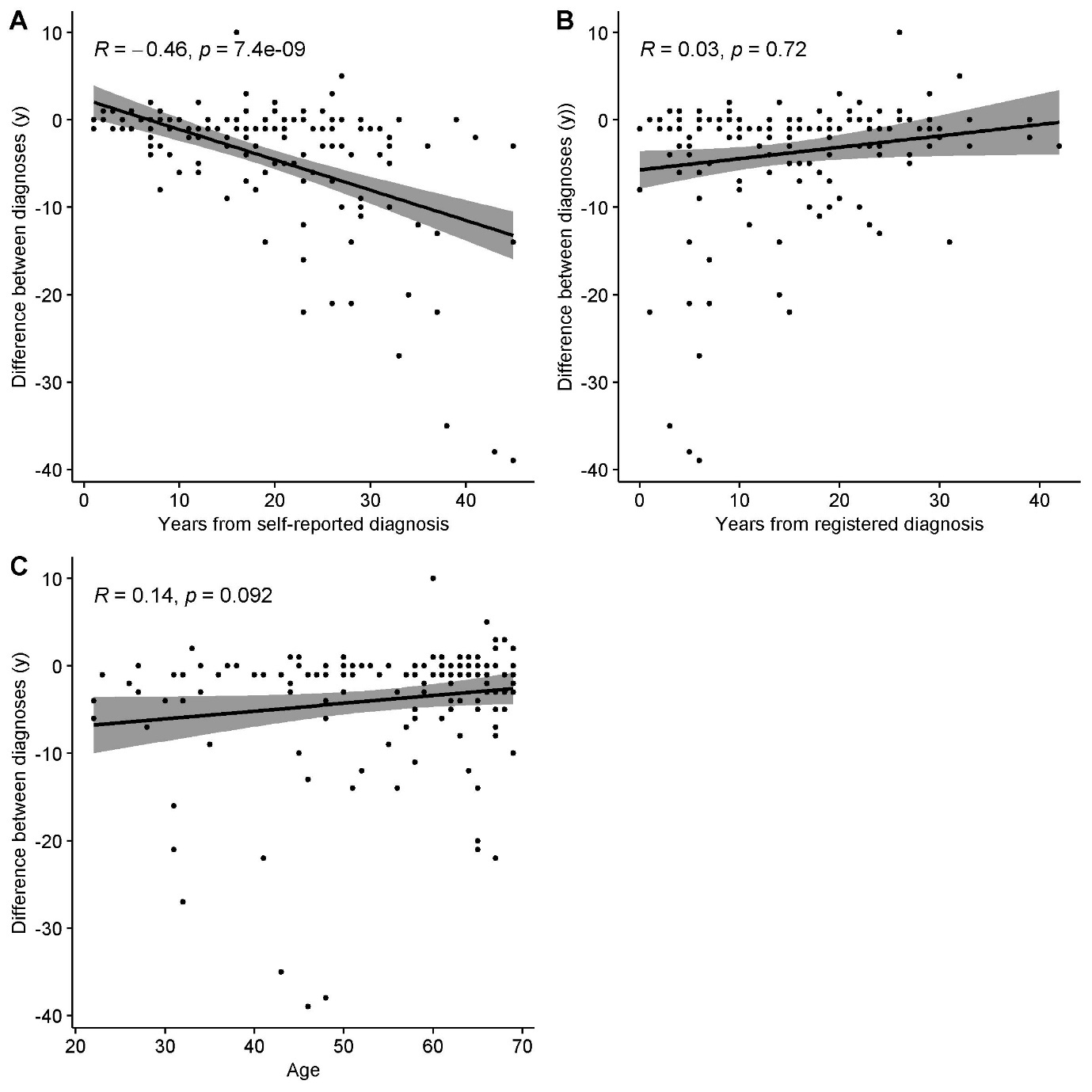
 **S-Figure 4.** Correlation between the time difference and years elapsed from self-reported physician-diagnosed asthma (A), between the time difference and years elapsed from registered asthma diagnosis (B) between the time difference and calendar age (C) in Western Finland. Spearman’s *r* and P-value of the correlation is shown.
